# Assessment of Carbon Sequestration in Private Forests across Two Different Physiographic Regions of Nepal: Implications for Conservation and Climate Change Mitigation

**DOI:** 10.1155/2023/6599067

**Published:** 2023-12-04

**Authors:** Rajeev Joshi, Tej Kumar Shrestha, Bijaya Mishra, Jeetendra Gautam, Bijay Maharjan, Kamal Raj Gosai, Tek Maraseni, Bijaya Neupane

**Affiliations:** ^1^College of Natural Resource Management, Faculty of Forestry, Agriculture and Forestry University, Katari 56310, Udayapur, Nepal; ^2^School of Natural Sciences, Massey University, Albany Campus, Palmerston North, New Zealand; ^3^Lumbini Environmental Services Pvt. Ltd., New Baneshwor, Kathmandu, Nepal; ^4^Faculty of Forestry, Agriculture and Forestry University, Hetauda, Nepal; ^5^Lumbini Environmental Services Pvt. Ltd., New Baneshwor, Kathmandu, Nepal; ^6^Tri-Chandra Multiple Campus, Ghantaghar, Kathmandu, Nepal; ^7^Institute for Life Sciences and the Environment, University of Southern Queensland, Toowoomba, QLD 4350, Australia; ^8^Department of Forest Sciences, Faculty of Agriculture and Forestry, University of Helsinki, Helsinki 00014, Finland

## Abstract

Private forests offer diverse ecosystem services, including carbon sequestration and biodiversity conservation, which are crucial for Nepal. However, there is a notable absence of comprehensive research on these services. Assessing carbon sequestration in private forests can have economic advantages for forest owners by promoting resource conservation and contributing to greenhouse gas reduction. This study aims to estimate and compare carbon stocks in private forests located in two distinct physiographic regions of Nepal while also identifying the factors influencing these carbon stocks. The analysis focuses on 16 private forests (with 0.1 to 0.5 hectares) each from Chitwan district (Terai region) and Kavrepalanchok district (Hilly region). Field data collection involved direct measurements of tree and sapling diameter at breast height (DBH), as well as height and class of trees and poles, utilizing a total enumeration method. These collected values were utilized to calculate aboveground biomass (AGTB), aboveground sapling biomass (AGSB), belowground biomass, and carbon stock. Private forests of Terai region were dominated by *Shorea borneensis*, *Tectona grandis*, and *Dalbergia sissoo*, whereas the Hilly region was dominated by *Pinus patula*, *Alnus nepalensis*, *Schima wallichii*, and *Quercus leucotrichophora*. The aboveground biomass carbon in the Terai region's private forests was estimated to be 83.53 t·ha^−1^, while in the Hilly region, it was 37.32 t·ha^−1^. The belowground biomass carbon in the Terai region's private forests was found to be 21.72 t·ha^−1^, compared to 9.70 t·ha^−1^ in the Hilly region. Consequently, the estimated total carbon stock in the Terai and Hilly regions' private forests was 105.25 t·ha^−1^ (386.26 t·ha^−1^ CO_2_-eq) and 47.02 t·ha^−1^ (172.57 t·ha^−1^ CO_2_-eq), respectively. Carbon sequestration in the Terai region's private forests was discovered to be 2.24 times higher than that in the Hilly region. These findings underscore the significant potential of private forests, which can generate economic benefits through carbon trading and leverage mechanisms such as REDD+/CDM to promote sustainable conservation practices.

## 1. Introduction

The significance of forested areas in carbon sequestration has already been well acknowledged and documented [1–3]. Carbon sequestration, which involves removing excess carbon from the atmosphere and depositing it primarily through changes in land use [4], has been recognized as a crucial process. Forests can effectively capture and store atmospheric carbon in both aboveground and belowground biomass through photosynthesis. Forests also have a vital role in the climate system [5], serving as both sources and sinks of carbon on a global scale [6, 7]. The growth of forests is believed to have significant potential for carbon sequestration and is considered a cost-effective approach to mitigating global climate change [8–10]. Consequently, REDD (Reducing Emissions from Deforestation and Forest Degradation) has evolved into REDD+, which integrates conservation efforts, sustainable forest management, and the enhancement of forest carbon stocks [11, 12]. In tropical countries, REDD+ is regarded as an effective and efficient mitigation mechanism for combating the impacts of climate change [13]. In Nepal, private forests also offer significant potential for carbon sequestration [14–16]. Therefore, private forests can be considered major carbon sinks and can play a significant role within the REDD+ scheme.

Numerous studies have demonstrated the effectiveness of well-managed forest areas in storing global carbon [10, 17, 18]. The private forestry program offers a more holistic approach that addresses livelihood concerns and mitigates environmental degradation through sustainable forest management [19, 20]. Private forests in Nepal are distinct from national forests as they are managed and owned by individuals on private land, in accordance with existing laws. Private forest owners have the autonomy to protect, develop, and manage their land, as well as utilize or sell forest products, within the framework of prevailing rules and regulations [21].

In Nepal, Prime Minister Juddha Sumsher initiated a policy requiring the planting of a sapling before felling a mature tree on private land, marking a significant moment in private forest development [22]. In 1957, the Private Forests Nationalization Act was enacted with the aim of nationalizing privately owned forests. This legislation-imposed ownership limits on private forests, allowing individuals to own a maximum of 25 ropani (1.3 ha) in the Hills or 5 bighas (3.4 ha) in the Terai. Unfortunately, this restriction discouraged tree planting on private lands, as farmers feared further limitations by the government. The extent and registration of private forests in the country are not promising. According to the Department of Forest's recent report, out of 77 districts, only 62 have records of private forests and their registration. A mere 3,753 private individuals have registered 2,902 ha of their forests as private forests at their respective District Forest Offices [23]. Data reveals that the Terai districts have a higher number of registered private forests compared to the Hilly districts [22]. In the Terai region, trees are primarily cultivated on private farms, while in the Hills, they typically grow naturally and are protected by farmers. Farmers now have the liberty to directly harvest, sell, and transport 23 common tree species, predominantly found on private lands [24]. Among these 23 species, *Dalbergia sissoo*, *Tectona grandis*, *Toona ciliata*, *Eucalyptus*species, *Anthocephalus cadamba*, and *Mangifera indica* are the most popular choices. Importantly, individual farmers are only required to visit the relevant forest office once to register, endorse their stock, and obtain permits for transporting the harvested timber. In Nepal, research efforts have predominantly focused on the tangible economic benefits of forests, with limited studies conducted on intangible benefits such as carbon sequestration and biodiversity conservation in private forests. Consequently, there is a lack of information regarding carbon stocks in private forest ecosystems in Nepal. Currently, there are diverse mechanisms for carbon trade, which can significantly contribute to increasing the income of forest owners/farmers. Consequently, there is a growing interest among forest users in understanding and harnessing the potential of carbon sequestration.

Nepal has made significant progress in the REDD+ readiness phase and has implemented the Emissions Reduction Program in thirteen districts of the Terai Arc Landscape in the Terai region [25, 26]. It is crucial to ensure that the implementation of the REDD+ program does not have adverse impacts on biodiversity and local communities [27]. In this context, the Biodiversity Monitoring Protocol (BMP) for REDD+ will play a pivotal role in guiding project proponents in selecting appropriate methodologies and formulating criteria and indicators while establishing the biodiversity baseline of the project area. In addition, the BMP will assist in reassessing changes in biodiversity following the implementation of REDD+ activities. The participation in the REDD+ mechanism holds promising prospects for Nepal as it provides opportunities for the country's forests to engage in carbon trading, generating carbon revenues and noncarbon benefits for the nation and its people. Preliminary estimates indicate that REDD+ may bring in between $20 and 86 million per year for Nepal [28]. Therefore, private forests in Nepal can serve as the most viable option for generating carbon revenues and noncarbon benefits for the country. This private forestry model can be a valuable concept in designing REDD+ policies and programs for stakeholders involved in private forest-based REDD+ initiatives in developing countries. By increasing the carbon sequestration rate of private forests, farmers/owners can gain financial benefits through carbon credits, while indigenous peoples who depend on the forest can maintain sustainable livelihoods and receive the associated benefits.

The estimation of carbon stocks is currently an important issue, but there is limited research on quantifying carbon stocks in private forests, highlighting the need for this study. Although a few studies have been conducted in accessible forest areas in Nepal, data from remotely located forest areas is still lacking. Therefore, this study on carbon stock estimation in private forests will provide baseline data for future research and contribute to the sustainable conservation and management of private forests. REDD+ has been piloted in developing countries as a climate change mitigation strategy, offering financial incentives for carbon sequestration in forests. This paper also investigates the viability of introducing REDD+ in privately‐owned forests across two physiographic regions in central Nepal: the Terai and Hilly regions, encompassing the Chitwan and Kavrepalanchok districts, respectively, by leveraging carbon sequestration data. This study aims to estimate and compare carbon stocks in private forests located in two distinct physiographic regions of Nepal and contribute to a comparative analysis of carbon sequestration in private forests. In addition, the study will offer information on the total aboveground biomass of dominant species in selected private forests, which can help in developing a payment scheme for carbon credits. These findings will inform local communities about the carbon status of vegetation in their specific private forests, serving as an indicator of climate change mitigation. Moreover, estimating and managing carbon stocks in private forests can be a potential strategy for greenhouse gas mitigation.

## 2. Materials and Methods

### 2.1. Study Area

**Figure 1:**
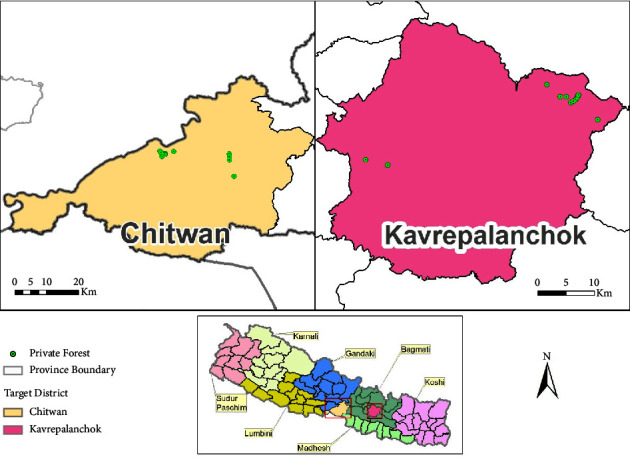
Selected districts for study area and private forest distribution.

The research was conducted in private forests located in two distinct physiographic regions of Nepal: the Terai region and the Hilly region. Specifically, the Chitwan district in the Terai region and the Kavrepalanchok districts in the Hilly region were chosen from the Bagmati Province (Figure 1). The Bagmati Province, situated in the central part of Nepal, spans between 27.6625°N latitude and 85.4376°E longitude. The Province's elevation ranges from 141 meters at Golaghat in Chitwan district to 7,422 meters at Ganesh Himal. A list of potential private forests was compiled from the respective districts, and a screening process took their sizes into consideration (ranging from 0.1 to 0.5 hectares). Eventually, 16 private forests were selected from each district. Moreover, for the Chitwan and Kavrepalanchok districts, two wards from different municipalities were randomly chosen as the locations for the private forests.

### 2.2. Data Collection

Prior to deploying the data collection team in the field, coordination and consultation were carried out with the relevant Divisional Forest Officers (DFO) in This paper also investigates the viability of introducing REDD+ in privately-owned forests across two physiographic regions in central Nepal: the Terai and Hilly regions, encompassing the Chitwan and Kavrepalanchok districts, respectively by leveraging carbon sequestration data and Kavrepalanchok to obtain potential lists of private forests and their contact details. The list of potential private forests in Chitwan (Terai) and Kavrepalanchok (Hilly) districts was acquired from the provided lists by the respective DFOs. This initial list underwent further screening based on criteria such as area size (0.1 to 0.5 hectares), distribution across different municipalities, and wards within each district. As a result, 32 private forests were selected, with 16 private forests chosen from each district for subsequent inventory (refer to Figure 1). Contact information for the private forests was gathered through communication with staff and owners of the private forests, utilizing various means of contact. Relevant literature, including acts, policies, and guidelines, was reviewed. This involved examining forest inventory-related strategies, policies, and guidelines from sources such as the Department of Forest Research and Survey (DFRS) and the Forest Resource Assessment (FRA). Prior to commencing fieldwork, the team received field forms, topographic maps, and necessary field equipment. The field forms contained essential forest information, such as the owner's name, area, registration date, GPS location, forest type, and condition. In addition, the forms included sections for recording measurements of saplings, trees, and poles. The inventory equipment's condition was checked and verified before initiating fieldwork. GPS files indicating the location of each inventory site within the inventory area were stored in the GPS unit.

### 2.3. Forest Sampling Design and Measurement

The estimation of aboveground carbon sequestration in private forests was conducted using the total enumeration method. This involved employing the total count method for trees and poles with a diameter at breast height (DBH) greater than 5 cm, as well as for saplings with a DBH ranging from 1 to 5 cm. Diameter at breast height (DBH) of each tree within the plot was measured using diameter tape at 1.3 m, and the height of each tree was determined using a Silva clinometer. The carbon stock was derived from the aboveground tree biomass (AGTB) and the aboveground saplings biomass (AGSB). Standard methods were applied to estimate each of these parameters.

#### 2.3.1. Aboveground Tree Biomass (AGTB)

The aboveground tree biomass in the private forests was estimated using the allometric equation developed by Chave et al. [29] specifically for moist forest stands. The equation used was AGTB = 0.0509 × *ρ* × *D*^2^ × *H*, where AGTB represents the aboveground tree biomass in kilograms (kg), *ρ* refers to the dry wood density in grams per cubic centimeter (gm·cm^−3^) [30], *D* represents the tree diameter at breast·height in centimeters (cm), and *H* represents the tree height in meters (m). The obtained biomass value was then multiplied by the default carbon fraction of 0.47 from the Intergovernmental Panel on Climate Change [6] to estimate the carbon content.

#### 2.3.2. Aboveground Saplings Biomass (AGSB)

In order to determine the aboveground sapling biomass (AGSB) in the private forests, a national allometric biomass table was employed. A regression model, represented by the given equation, was utilized for various species to calculate the biomass. The regression model is expressed as Log (AGSB) = *a* + *b* log (*D*), where log represents the natural logarithm (dimensionless), AGSB denotes the aboveground sapling biomass measured in kilograms (kg), *a* represents the intercept of the allometric relationship for saplings (dimensionless), *b* represents the slope of the allometric relationship for saplings (dimensionless), and *D* represents the over bark diameter at breast·height, which is measured at 1.3 meters above ground, in centimeters (cm). To convert the biomass stock densities into carbon stock densities, the default carbon fraction of 0.47 from the Intergovernmental Panel on Climate Change [6] was used.

#### 2.3.3. Belowground Biomass (BGB)

Measuring belowground biomass, specifically the biomass of roots, is a challenging and time-consuming task compared to measuring aboveground biomass. It is also highly uncertain. The calculation of belowground biomass involved multiplying the value of aboveground biomass (AGB) by the constant factor of 0.26, as recommended by the Good Practice Guidelines (GPG) of the Intergovernmental Panel on Climate Change [6], Mandal and Joshi [4], and Pandey et al. [10, 18]. The equation used for this calculation is as follows: BGB = AGB^*∗*^0.26, where BGB represents the belowground biomass and AGB represents the aboveground biomass.

#### 2.3.4. Total Carbon Stock

The carbon values of aboveground tree biomass (AGTB) and aboveground sapling biomass (AGSB) were added together to calculate the total aboveground carbon stock. To estimate the total forest carbon stock, the following equation was utilized:

(1) $$\begin{array}{c} \text{TC}=C\left(\text{AGTB}\right)+C\left(\text{AGSB}\right)+C\left(\text{BGTB}\right)+C\left(\text{BGSB}\right). \end{array}$$

In this equation:

1. TC represents the total forest carbon stock measured in metric tons per hectare (t·ha^−1^)
2. *C* (AGTB) represents the carbon stock in aboveground tree biomass measured in metric tons per hectare (t·ha^−1^)
3. *C* (AGSB) represents the carbon stock in aboveground sapling biomass measured in metric tons per hectare (t·ha^−1^)
4. *C* (BGTB) represents the carbon stock in belowground tree biomass measured in metric tons per hectare (t·ha^−1^)
5. *C* (BGSB) represents the carbon stock in belowground sapling biomass measured in metric tons per hectare (t·ha^−1^)

#### 2.3.5. Carbon-Dioxide Equivalent (CO_2_-Eq)

The total forest carbon stock was transformed into CO_2_-eq by multiplying it by 44/12, which is equal to 3.67, as recommended by Pearson et al. [31].

### 2.4. Data Analysis

The gathered data were analyzed using Microsoft Excel software to obtain the necessary attributes and gather additional information on the carbon stock of private forests in two distinct physiographic zones. In addition, the required graphs for the analysis were generated using the same software. A comparison was conducted between the biomass and carbon content of two categories, i.e., private forests in Chitwan and private forests in Kavrepalanchok. The comparison was performed at three levels: tree, sapling, and total (tree and sapling). Statistical analysis was conducted using R version 4.3.0 and R Studio version 2023.06.0.

#### 2.4.1. Statistical Analysis

We conducted a two-sample independent *t*-test to assess the significant difference in aboveground biomass and carbon between Terai and Hilly private forests, using a 95% confidence level (*α* = 0.05). After excluding outliers from both regions' total samples, we had 14 samples from Terai and 13 samples from Hill private forests for the *t*-test on aboveground tree and sapling biomass and carbon. In addition, we analyzed 13 samples from Terai private forests and 13 samples from Hilly private forests using the *t*-test for total aboveground biomass. Furthermore, we conducted a *t*-test on 11 samples from Terai private forests and 15 samples from Hill private forests to evaluate total aboveground carbon content.

The null hypothesis (*H*_0_) stated that the true difference in means between tree biomass and carbon of private forests in Terai and Hill is equal to 0, while the alternative hypothesis (*H*_1_) stated that the true difference in means is not equal to 0. To delve deeper into this assertion, we performed a one-tailed (greater) *t*-test under the same statistical conditions as mentioned earlier. In this case, the null hypothesis (*H*_0_) remained unchanged, while the alternative hypothesis (*H*_1_) asserted that the true difference in means between aboveground biomass and carbon of private forests in Terai and Hill is greater than zero.

## 3. Results

### 3.1. Vegetation Parameters

Vegetation analysis was conducted on the private forests in both the Terai and Hill regions, considering various parameters. A total of 35 tree species were identified and recorded, with 24 species found in the Hill region and 11 species in the Terai belt. In the private forests of the Terai region, 11 tree species were identified, with a total of 679 stems per hectare measured. Similarly, in the private forests of the Hill region, 24 tree species were identified, with a total of 576 stems per hectare measured. Consequently, a higher tree density of 679 stems per hectare was observed in the private forests of the Terai region, followed by 576 stems per hectare in the private forests of the Hill region (Table 1).

The prominent tree species found in the private forests of the Terai region include *Tectona grandis*, *Shorea borneensis*, *Dalbergia sissoo*, *Swietenia mahagoni*, *Bombax ceiba*, *Melia azedarach*, *Artocarpus lacucha*, *Bauhinia purpurea*, *Neolamarckia cadamba*, and others (Table 2). Similarly, the principal tree species found in the private forests of the Hill region include *Pinus patula*, *Alnus nepalensis*, *Quercus leucotrichophora*, *Schima wallichii*, *Myrica esculenta*, *Prunus cerasoides*, *Castranopsis hystrix*, *Pinus wallichiana*, *Tsuga dumosa*, and others (Table 3). No common tree species were identified between the study areas of the Terai and Hill regions, possibly due to the differences in the physiographic regions of the country.

### 3.2. Aboveground Tree Biomass (AGTB) and Carbon Stock

**Figure 2:**
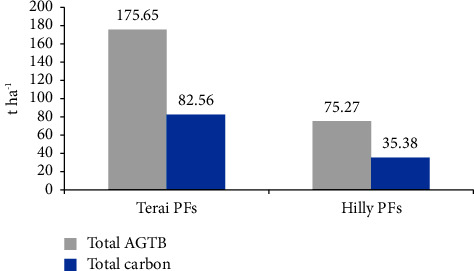
Total AGTB and carbon content in both PFs.

In the private forests of the Hilly region, the aboveground tree biomass (AGTB) was measured to be 75.27 t·ha^−1^, and the carbon stock was 35.38 t·ha^−1^, which was lower compared to the private forests of the Terai region where the biomass was 175.65 t·ha^−1^ and carbon stock was 82.56 t·ha^−1^. The dominant aboveground tree biomass was observed in the private forests of the Terai region, mainly due to the presence of larger-sized trees, which naturally have higher biomass values (Figure 2).

**Figure 3:**
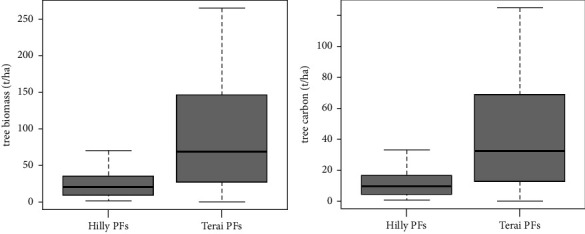
Boxplot showing the comparison of aboveground tree biomass (t·ha^−1^) and carbon (t·ha^−1^) between both PFs.

The findings reveal a *p* value of 0.01083, which is below the significance level of 0.05, indicating a notable difference in aboveground tree biomass and carbon content between the private forests of the Terai and Hill regions. Moreover, the boxplot graphically illustrates that the aboveground tree biomass and carbon values are notably greater in the private forests of the Terai in comparison to the Hill region. Furthermore, the results of the one-tailed *t*-test generated *p* values of 0.005415 for biomass and 0.004498 for carbon, both of which fall below the 0.05 significance threshold. This underscores that the aboveground tree biomass and carbon content in Terai private forests are significantly higher than in the private forests of the Hill region (see Figure 3).

### 3.3. Aboveground Sapling Biomass (AGSB) and Carbon Stock

**Figure 4:**
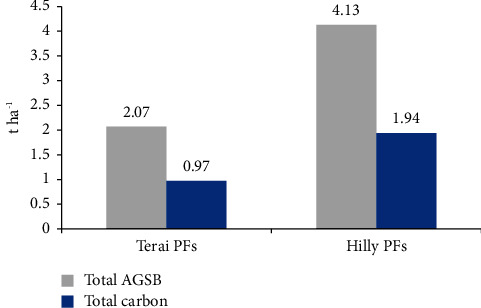
Total AGSB and carbon content in both PFs.

The estimated aboveground sapling biomass (AGSB) and carbon stock in the private forests of the Terai region were found to be 2.07 t·ha^−1^ and 0.97 t·ha^−1^, respectively, which were lower compared to the private forests of the Hilly region where the biomass was 4.13 t·ha^−1^ and carbon stock was 1.94 t·ha^−1^. The dominant aboveground sapling biomass was observed in the private forests of the Hilly region due to the higher number of saplings present, resulting in higher biomass values (Figure 4).

**Figure 5:**
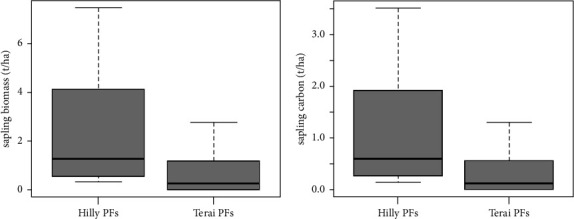
Boxplot showing the comparison of aboveground sapling biomass (t·ha^−1^) and carbon (t·ha^−1^) between both PFs.

The findings displayed a *p* value of 0.03485, falling below the 0.05 significance level, indicating a noteworthy distinction in aboveground sapling biomass and carbon content between the private forests of the Terai and the Hills. Furthermore, the boxplot illustrated that the aboveground sapling biomass and carbon values are less in the private forests of the Terai in contrast to those in the Hill region. Similarly, the outcome of the one-tailed *t*-test yielded a *p* value of 0.01742, which is below the 0.05 threshold. This implies that the aboveground sapling biomass and carbon content in Terai private forests is significantly lesser than in the private forests of the Hill region (refer to Figure 5).

### 3.4. Total Aboveground Biomass (AGB) and Carbon Stock

**Figure 6:**
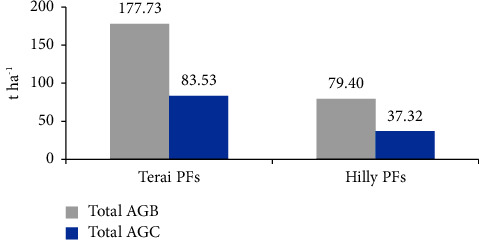
Total AGB and carbon content in both PFs.

The majority of the carbon stock in Terai region was contributed by aboveground tree carbon (AGTC) with a value of 82.56 t·ha^−1^, followed by aboveground sapling carbon (AGSC) with 0.97 t·ha^−1^. Therefore, the total carbon stock was estimated to be 83.53 t·ha^−1^ (Figure 6). Similarly, AGTC accounted for the highest contribution with 35.38 t·ha^−1^, followed by AGSC with 1.94 t·ha^−1^ in Hilly region. Hence, the estimated total aboveground carbon stock was 37.32 t·ha^−1^ (Figure 6).

The results indicated a *p* value of 0.008996, which falls below the 0.05 significance level, signifying a substantial disparity in the total biomass and carbon values between the private forests of the Terai and the Hill region. In addition, the boxplot illustrated that the total biomass and carbon values are higher in the private forests of the Terai when compared to those in the Hill region.

**Figure 7:**
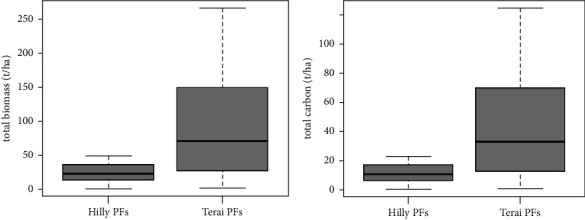
Boxplot showing the comparison of total aboveground biomass (t·ha^−1^) and carbon (t·ha^−1^) between both PFs.

Similarly, the outcome of the one-tailed *t*-test produced a *p* value of 0.004498, which is less than 0.05. This indicates that the total aboveground biomass and carbon values in Terai private forests are significantly greater than in the private forests of the Hill region (see Figure 7).

### 3.5. Belowground Tree Biomass (BGTB) and Carbon Stock

The accumulation of belowground tree biomass (BGTB) and the corresponding carbon stock in the private forests of the Terai region were estimated to be 45.67 t·ha^−1^ and 21.46 t·ha^−1^, respectively. On the other hand, in the private forests of the Hill region, the BGTB accumulation and carbon stock were estimated to be 19.57 t·ha^−1^ and 9.20 t·ha^−1^, respectively. Consequently, the private forests of the Terai region exhibited higher levels of BGTB accumulation and carbon stock compared to the private forests of the Hill region (Table 4).

### 3.6. Belowground Sapling Biomass (BGSB) and Carbon Stock

The accumulation of belowground sapling biomass (BGSB) and the corresponding carbon stock in the private forests of the Terai region were estimated to be 0.54 t·ha^−1^ and 0.26 t·ha^−1^, respectively. In comparison, the BGSB accumulation and carbon stock in the private forests of the Hilly region were estimated to be 1.07 t·ha^−1^ and 0.50 t·ha^−1^, respectively. As a result, the private forests of the Terai region exhibited lower levels of BGSB accumulation and carbon stock compared to the private forests of the Hilly region (as indicated in Table 4).

### 3.7. Total Belowground Biomass (BGB) and Carbon Stock

**Figure 8:**
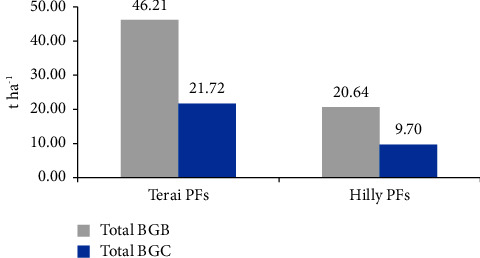
Total BGB and carbon content in both PFs.

The accumulation of belowground biomass (BGB) and the corresponding carbon stock in the private forests of the Terai region were estimated to be 46.21 t·ha^−1^ and 21.72 t·ha^−1^, respectively. Similarly, the BGB accumulation and carbon stock in the private forests of the Hilly region were estimated to be 20.64 t·ha^−1^ and 9.70 t·ha^−1^, respectively. As a result, the private forests of the Terai region exhibited higher levels of BGB accumulation and carbon stock compared to the private forests of the Hilly region (as depicted in Figure 8).

### 3.8. Total Carbon Stock

The estimated total carbon stock in the private forests of the Terai region was 105.25 t·ha^−1^. The largest contribution to the carbon stock came from aboveground tree carbon (82.56 t·ha^−1^), followed by belowground tree carbon (21.46 t·ha^−1^), as indicated in Table 4. Similarly, the total carbon stock in the private forests of the Hilly region was estimated to be 47.02 t·ha^−1^. The primary contributor to the carbon stock in this region was aboveground tree carbon (35.38 t·ha^−1^), followed by belowground tree carbon (9.20 t·ha^−1^) (Table 4).

### 3.9. Carbon-Dioxide Equivalent

**Figure 9:**
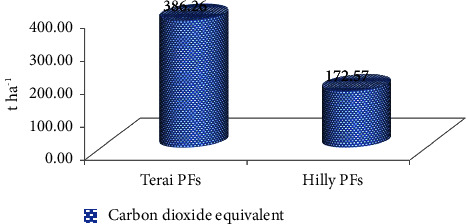
Carbon-dioxide equivalent in PFs.

The carbon dioxide equivalent in the private forests of the Terai and Hill regions was estimated to be 386.26 t·ha^−1^ and 172.57 t·ha^−1^, respectively, (as illustrated in Figure 9).

## 4. Discussion

### 4.1. Vegetation Parameters

A total of 35 species were documented in both private forests (PFs), with a greater variety of species found in the Hill region compared to the Terai region. This disparity could be attributed to the favorable forest conditions and local factors influencing temperature regimes and microclimates [32]. The Terai region, being relatively drier, exhibited a decreasing trend in the total number of species. The decline in species diversity in the Terai, along with the presence of a small number of unique species, may indicate the impact of repeated exploitation on specific species [33]. Excessive degradation and disturbances in private forests can lead to the loss of late successional species while favoring early successional and disturbance-tolerant species [34].

Higher tree density was observed in the private forests of the Terai region (679 stems/ha) compared to the Hill region (576 stems/ha). The FRA report (2015) states that Terai forests in Nepal have an average of 583.40 stems/ha (≥5 cm DBH), which is lower than the findings of our study. Similarly, the Churia belt in Nepal has an average of 731 trees/ha (≥5 cm DBH), which is higher than our study's results. In addition, the mean diameter at breast height (DBH) and height of tree species were higher in the private forests of the Terai region than in the Hill region, potentially due to the presence of a greater number of mature species in Terai's private forests. In contrast, the trees and poles in the Hill region were slightly smaller in height, possibly a result of harsher environmental conditions and human disturbances [35, 36]. This difference in tree characteristics aligns with the distinction in forest effectiveness for carbon sequestration and biodiversity support between Terai and hill private forests, likely influenced by region-specific climatic factors. In addition, the prevalence of an integrated farming system relying on livestock fodder in hilly areas contrasts with the mechanization prevalent in the Terai region (as indicated in Table 1). Moreover, high-altitude areas experience lower temperatures, restricting plant growth, while low-altitude areas face insufficient rainfall and areunable to meet moisture requirements for plant growth [37]. The optimal range of temperature and precipitation tends to occur in middle elevations [38].

### 4.2. Total Aboveground Biomass (AGB) and Carbon Stock

Biomass levels in different plots of the same community forests and among different community forests vary due to variations in tree age, size, forest composition, and tree density. Trees have the ability to develop significant biomass and capture a large amount of carbon over many decades. Consequently, forests can serve as effective carbon sinks and store carbon for extended periods. The carbon sink and storage capacity in forests are interdependent. In the studied private forests, many trees had a diameter at breast height (DBH) of less than 20 cm. According to Johnson and Coburn's [39], well-stocked forests typically sequester carbon at the highest rate between the ages of approximately 10 and 20–30. As an indicator, forests with varying productivity levels can sequester about 200–520 tons of carbon dioxide (CO_2_-eq) per hectare by the age of 30 years [39]. Thus, the studied private forests have the potential to sequester more carbon. Among the studied private forests, the Terai region exhibited higher aboveground biomass and carbon stock compared to the Hill region. This dominance in aboveground tree and sapling biomass in the Terai region can be attributed to the presence of larger-sized trees and saplings, which naturally have higher biomass values. The Terai region's private forest mainly consists of tree and sapling stands, including species such as *Tectona grandis*, *Shorea borneensis*, *Dalbergia sissoo*, *Swietenia mahagoni*, *Bombax ceiba*, *Melia azedarach*, *Artocarpus lacucha*, *Bauhinia purpurea*, and *Neolamarckia cadamba*, among others, with high wood densities. This study is consistent with the carbon assessment conducted by Jati [40] in the Kumvakarna Conservation Community Forest, KCAP, Taplejung, which found tree biomass carbon to be 109.10 t·ha^−1^ in preserved forest (PF) and 177.44 t·ha^−1^ in managed forest (MF).

The total aboveground biomass of both PFs falls within the range of aboveground biomass observed in Indian forests (14–210 t·ha^−1^) estimated by Rabha [41]. However, the AGB estimation of both PFs was lower than the study conducted by Madugundu et al. [42], which reported a mean AGB of 280 ± 72.5 t·ha^−1^ based on ground data and 297.6 ± 55.2 t·ha^−1^ based on remote sensing in deciduous forests of the Western Ghats. Similarly, the AGB of the Hilly region (38.72 t·ha^−1^) was considerably lower than the AGB (100–160 t·ha^−1^) estimated by IPCC [6] for subtropical forests in the Asian continental region, whereas the AGB of the Terai region (109.17 t·ha^−1^) falls within the same range as the IPCC [6] estimates. The AGB of the studied PFs is lower than the AGB of Sal plantations in Meghalaya (406 t·ha^−1^) and Sal forests in the Satpura plateau (154.94 t·ha^−1^) as reported by Rabha [41]. Furthermore, according to the FRA report (2015), the Terai Forest contains 123.14 t·ha^−1^ of carbon, excluding seedlings, saplings of tree species, shrub species with less than 5 cm DBH, climbers, fine roots, grasses (including bamboos), and herbs, which is higher than the findings of the present study. In the Churia belt, the total carbon stock was estimated to be 116.94 t·ha^−1^, with tree components accounting for 84.73 t·ha^−1^, litter/debris contributing 0.31 t·ha^−1^, and soil containing 31.90 t·ha^−1^ of carbon. These values collectively exceed the findings of our study. The variation in aboveground biomass and carbon stock among these forests can be attributed to factors such as differences in forest age, forest type, site conditions, geographical regions, and local factors. In addition, the aboveground biomass of the vegetation is influenced by the diameter and age of trees and saplings.

The results indicate that the highest aboveground biomass (AGB) was observed at lower altitudes (Terai region) compared to higher altitudes (Hilly region). Numerous studies conducted in alpine ecosystems suggest that plant growth at higher altitude is primarily linked to temperature rather than precipitation [43]. Our findings, depicting a decline in AGB with increasing altitude, align with the results reported by Bhandari and Zhang [44]; Rauniyar et al. [45]; and Roukos et al. [46]. This trend may be attributed to two factors: first, the lower air temperature in the higher altitude zone resulting from reduced radiation input compared to other locations [47]; second, the pronounced impact of altitude on snowpack accumulation, affecting growing season length, soil water availability, and plant distribution more significantly at higher altitude sites than at lower ones. In summary, these findings suggest a detrimental impact of altitude on aboveground biomass.

### 4.3. Total Belowground Biomass (BGB) and Carbon Stock

A study conducted by Karky and Skutsch [14] on the belowground biomass of Namuna Community Forest in Illam found it to be 13.54 t·ha^−1^. Similarly, the belowground biomass (BGB) measured in the private forests of the Terai and Hill regions was 21.72 t·ha^−1^ and 9.70 t·ha^−1^, respectively. This indicates that the private forests in the Terai region have a higher biomass, while those in the Hill region have a lower biomass compared to Namuna Community Forest. However, another study by Dhakal [48] in the naturally regenerated forest of Pashupati Community Forest in Sarlahi reported a higher BGB of (181.83 ± 26.34) t·ha^−1^, exceeding the values observed in our study. Similarly, Joshi et al. [49] estimated the belowground biomass in degraded and nondegraded community forests of Nepal to be 43.31 t·ha^−1^ and 97.21 t·ha^−1^, respectively, which is higher than our findings in the private forests. The lower carbon stock in the private forests may be attributed to the younger trees and lower tree density.

### 4.4. Total Carbon Stock

Although limited studies have been conducted on carbon stock in private forests of Nepal, a study by Bhatta [50] comparing natural forests and community/private forests in the mixed broadleaf forests of Phulchoki watershed showed that natural forests had higher carbon storage. It was suggested that the low carbon content in community/private forests could be attributed to higher human consumption and encroachment. In addition, Jati [40] conducted a comparative study of carbon assessment in preserved and managed forests of Kumvakarna Conservation Community Forest (KCAP) in Taplejung, and the results indicated that managed forests (109.10 t·ha^−1^) had higher carbon storage compared to preserved forests (177.44 t·ha^−1^). It was concluded that managed forests were more efficient in carbon storage, despite facing disturbances such as fuel-wood collection, grazing, timber harvesting, and fodder collection. All these studies demonstrate higher carbon content than the current study in private forests. Similarly, Nizami [51] reported mean carbon stocks in subtropical managed and unmanaged forests of Pakistan, estimating carbon content to be 114 ± 2.26 t·ha^−1^ and 27.77 ± 1.66 t·ha^−1^, respectively, with managed forests showing higher carbon stock and unmanaged forests showing lower carbon stock compared to the studied private forests. Likewise, ANSAB [52] estimated carbon stock in Shorea robusta mixed subtropical Hill deciduous forest in Ludikhola of Gorkha to be between 165.91 t·ha^−1^ and 216.16 t·ha^−1^, which is comparatively higher than our study. Similarly, in their 2020 study, Joshi and colleagues found that the total carbon stock was 152.68 ± 22.95 t·ha^−1^ in degraded community forests and 301.08 ± 27.07 t·ha^−1^ in nondegraded community forests, which exceeded the levels observed in our current research. In addition, they reported that the CO_2_-eq measurement in both degraded and nondegraded community forests, reached 553 t·ha^−1^ and 1105 t·ha^−1^, respectively, which was approximately three times higher than our findings. These studies suggest that private forests hold significant potential and can yield economic benefits through carbon trading, taking advantage of the REDD+/CDM mechanism to promote sustainable private forest conservation.

## 5. Conclusion

The study revealed a significant difference in the total aboveground carbon stock density between private forests in the Terai and Hilly regions. The carbon sequestration also showed a notable contrast in biomass and carbon sequestration between the two regions. Specifically, the aboveground tree biomass was higher in the private forests of the Terai region, while the aboveground sapling biomass was higher in the private forests of the Hilly region. The findings provide evidence of the strong correlation between carbon stock and well-managed private forests, offering substantial potential for economic benefits through carbon trading under the Clean Development Mechanism (CDM), thereby promoting sustainable forest conservation. Carbon trading serves as a promising solution in the battle against global warming. However, implementing REDD+ in private forests presents both opportunities and challenges to households, with long-term policy implications. This is due to the differences in underlying concepts and frameworks between private forestry and REDD+, as the former focuses on forest product supply while the latter emphasizes incentivizing carbon sequestration in forests. The REDD+ mechanism can facilitate the development of other emission reduction programs to contribute to emission reduction targets. The results of this study can guide the design of REDD+ policies and programs for stakeholders involved in private forest-based REDD+ initiatives in developing countries.

### 5.1. Implications

Research on carbon stock and sequestration in Nepal has predominantly focused on forests, with limited studies conducted on quantifying carbon sequestration in private forests of different physiographic regions. Therefore, it is crucial for the government to encourage studies on forest composition, forest conditions (including degradation), and carbon-related issues in private forests. The global discussion and debate surrounding climate change and its impact on human lives have emphasized the significance of private forests as both carbon sinks and sources. However, the lack of comprehensive information on biomass and carbon stock in private forests hinders an accurate estimation of their overall contribution to carbon sequestration. To enhance viable atmospheric carbon sinks and mitigate climate change, the following recommendations should be implemented:

1. Sufficient research should be conducted encompassing different forest types, climatic zones, soil types, and forest management systems to gather comprehensive data on carbon stock and sequestration.
2. Remote sensing techniques combined with limited field data should be utilized to cost-effectively assess forest resources over large areas within a shorter timeframe.
3. The use of allometric equations specific to different tree species and ages is recommended for precise and accurate calculation of biomass in forest ecosystems.
4. Emphasis should be placed on selecting and planting species with higher carbon storage capacity across all forest types.
5. Since carbon content varies among different species, it is important to assess species-wise carbon content.
6. Effective forest management is highly recommended as it has significant potential for increased carbon sequestration, benefiting various forest user groups, particularly if forests are included under the Clean Development Mechanism.
7. Afforested and reforested forests exhibit higher carbon sequestration rates compared to older forests. Therefore, it is strongly recommended to undertake plantation efforts on barren land to regulate climatic conditions and reap the benefits of carbon trading.
8. Economic valuation of carbon sequestration must be conducted, and public awareness should be raised regarding the benefits of carbon sequestration.

## Figures and Tables

**Table 1:** Vegetation parameters with variation in PFs sties.

S.N	Parameters	Terai region (PF)	Hilly region (PF)
1	Number of species	11	24
2	Tree density (stems/ha)	679	576
3	Mean tree (DBH/cm)	17	11
4	Mean tree (height/m)	14	10
5	Sapling (number/ha)	94	102

**Table 2:** List of tree species observed in Terai region.

S. No.	Botanical name	Vernacular name	Family	Frequency (%)
1	*Tectona grandis*	Teak	*Lamiaceae*	56
2	*Shorea borneensis*	Malaysian Sal	*Dipterocarpaceae*	56
3	*Dalbergia sissoo*	Sissoo	*Leguminosae*	25
4	*Swietenia mahagoni*	Mahogany	*Meliaceae*	19
5	*Bombax ceiba*	Simal	*Bombacaceae*	19
6	*Melia azedarach*	Bakaino	*Meliaceae*	44
7	*Artocarpus lacucha*	Badahar	*Moraceae*	6
8	*Bauhinia purpurea*	Taanki	*Fabaceae*	6
9	*Neolamarckia cadamba*	Kadam	*Rubiaceae*	6
10	*Albizia lebbeck*	Kalo Siris	*Fabaceae*	6
11	Unidentified spp	—	—	6

**Table 3:** List of tree species observed in Hilly region.

S. No.	Botanical name	Vernacular name	Family	Frequency (%)
1	*Pinus patula*	Pate salla	*Pinaceae*	69
2	*Alnus nepalensis*	Uttis	*Betulaceae*	69
3	*Rhododendron* spp	Gurans	*Ericaceae*	44
4	*Quercus leucotrichophora*	Banj	*Fagaceae*	31
5	*Schima wallichii*	Chilaune	*Theaceae*	38
6	*Myrica esculenta*	Kafal	*Myricaceae*	44
7	*Prunus cerasoides*	Paiyun	*Rosaceae*	69
8	*Castranopsis hystrix*	Katus	*Facaceae*	50
9	*Taxus wallichiana*	Lauth salla	*Taxaceae*	13
10	*Betula alnoides*	Saur	*Butulaceae*	6
11	*Sauraria nepalensis*	Gogan	*Saurauiaceae*	6
12	*Brassaiopsis hainla*	Chuletro	*Araliaceae*	6
13	*Pinus wallichiana*	Blue Pine	*Pinaceae*	19
14	*Tsuga dumosa*	Himalayan Hemlock	*Pinaceae*	6
15	*Azadirachta indica*	Neem	*Meliaceae*	6
16	*Cinnamomum tamala*	Tejpat	*Lauraceae*	6
17	*Choerospondias axillaris*	Lapsi	*Anacardiaceae*	6
18	*Rhus semialata*	Bhakiamilo	*Anacardiaceae*	6
19	*Rhus succedanea*	Bhalayo	*Anacardiaceae*	6
20	*Magnolia champaka*	Chap	*Magnoliaceae*	6
21	*Osbeckia nepalensis*	Angeri	*Melastomataceae*	13
22	*Pyrus pashia*	Mel	*Rosaceae*	6
23	*Ficus nemoralis*	Dhudhilo	*Moraceae*	19
24	*Morus alba*	Kimbu	*Moraceae*	6

**Table 4:** Total biomass and carbon in different pools of both PFs.

S. No.	Carbon pools	Terai PFs		Hilly PFs	
		Biomass (t·ha^−1^)	Carbon (t·ha^−1^)	Biomass (t·ha^−1^)	Carbon (t·ha^−1^)
1	Aboveground trees	175.65	82.56	75.27	35.38
2	Aboveground saplings	2.07	0.97	4.13	1.94
3	Belowground trees	45.67	21.46	19.57	9.20
4	Belowground saplings	0.54	0.26	1.07	0.50
Total		**223.93**	**105.25**	**100.04**	**47.02**

The results indicated a *p* value of 0.008996, which falls below the 0.05 significance level, signifying a substantial disparity in the total biomass and carbon values between the private forests of the Terai and the hilly region.

## Data Availability

The data used to support the findings of this study are available from the corresponding author upon reasonable request.
